# GTV-based prescription in SBRT for lung lesions using advanced dose calculation algorithms

**DOI:** 10.1186/s13014-014-0223-5

**Published:** 2014-10-16

**Authors:** Thomas Lacornerie, Albert Lisbona, Xavier Mirabel, Eric Lartigau, Nick Reynaert

**Affiliations:** Service de Physique Médicale, Centre Oscar Lambret, Lille, France; Service de Physique Médicale, Institut de Cancérologie de l’Ouest, Nantes, France; Département Universitaire de Radiothérapie, Centre Oscar Lambret, Lille, France

## Abstract

**Background:**

The aim of current study was to investigate the way dose is prescribed to lung lesions during SBRT using advanced dose calculation algorithms that take into account electron transport (type B algorithms). As type A algorithms do not take into account secondary electron transport, they overestimate the dose to lung lesions. Type B algorithms are more accurate but still no consensus is reached regarding dose prescription. The positive clinical results obtained using type A algorithms should be used as a starting point.

**Methods:**

In current work a dose-calculation experiment is performed, presenting different prescription methods. Three cases with three different sizes of peripheral lung lesions were planned using three different treatment platforms. For each individual case 60 Gy to the PTV was prescribed using a type A algorithm and the dose distribution was recalculated using a type B algorithm in order to evaluate the impact of the secondary electron transport. Secondly, for each case a type B algorithm was used to prescribe 48 Gy to the PTV, and the resulting doses to the GTV were analyzed. Finally, prescriptions based on specific GTV dose volumes were evaluated.

**Results:**

When using a type A algorithm to prescribe the same dose to the PTV, the differences regarding median GTV doses among platforms and cases were always less than 10% of the prescription dose. The prescription to the PTV based on type B algorithms, leads to a more important variability of the median GTV dose among cases and among platforms, (respectively 24%, and 28%). However, when 54 Gy was prescribed as median GTV dose, using a type B algorithm, the variability observed was minimal.

**Conclusion:**

Normalizing the prescription dose to the median GTV dose for lung lesions avoids variability among different cases and treatment platforms of SBRT when type B algorithms are used to calculate the dose. The combination of using a type A algorithm to optimize a homogeneous dose in the PTV and using a type B algorithm to prescribe the median GTV dose provides a very robust method for treating lung lesions.

**Electronic supplementary material:**

The online version of this article (doi:10.1186/s13014-014-0223-5) contains supplementary material, which is available to authorized users.

## Background

The differences between radiotherapy dose calculation algorithms that take into account the electron transport phenomenon inside non-homogeneous environments (category 3&4 or type B) and those that do not (category 1&2 or type A) are well known [1]. The clinical implications of Monte-Carlo dose calculation algorithms [2,3] have been described, as well. However, the differences between type A and type B algorithms for small fields – with a penumbra region that occupies most of the field area – depend on many factors, such as lung density, beam sizes, and the position and size of the target. It is still not clearly understood how to adapt the protocols based on type A algorithms. Xiao et al. [4] concluded their study about heterogeneity corrections and RTOG trial 0236 in 2009 stating *“the information provided by the study will be used for future protocols,”*. But almost 3 years later Li et al. stated, about heterogeneities and the RTOG trial 0813 [5], that “*further studies are expected to establish protocol criteria for MC dose calculations*”. The RTOG trial 0915 requires the use of a type B algorithm with a PTV edge prescription and an isodose between 60 and 90% of the dose maximum. That variability leads to significantly different GTV doses as we will demonstrate. Many articles have pointed out the discrepancies between type A and type B algorithms in this journal [6-8] and in others for many years [9] but they are almost always focused on results for the PTV. We believe that the PTV edge prescription is not a good approach to be consistent between cases and platforms as will be shown below.

Discrepancies between Monte-Carlo (a type B algorithm) and Ray-Tracing algorithms (a type A “equivalent path length” algorithm) were monitored at the beginning of our experience with Cyberknife (Accuray Inc, Sunnyvale, CA) five years ago, although the protocol applied to peripheral lesions for dose prescription was the standard protocol defined by other teams for: 60 Gy in three fractions encompassing the PTV [10,11] calculated with type A algorithms.

**Figure 1:**
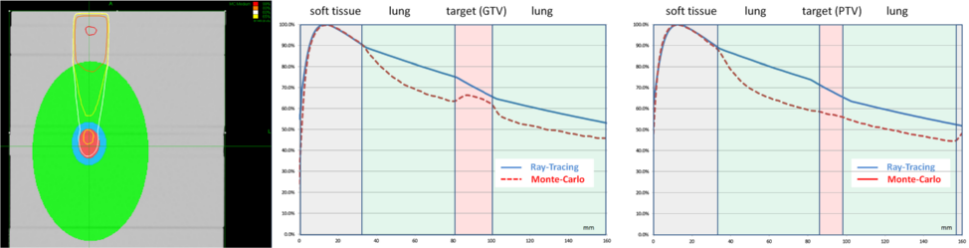
**PDD for 30 mm collimator calculated with Ray-Tracing and Monte-Carlo algorithms in phantom with different densities (left) on the beam axis (central) and 1 cm off axis (right), GTV (red, density = 1), PTV (blue), lung (green, density = 0.3).**

Because the Cyberknife has a 6-MV output and no flattening filter, the range of secondary electrons in lung can be up to 4 cm. In Figure 1, differences between Ray-Tracing and Monte-Carlo for a 30–mm beam in a phantom representing a 14-mm peripheral lung lesion is illustrated. Similar effects between any type A and type B algorithm can be observed.

**Figure 2:**
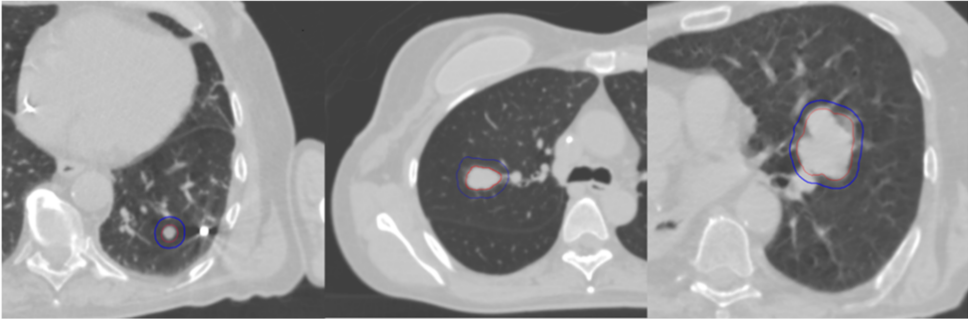
**GTV and PTV for the 3 different lesions analyzed.**

Ray-Tracing overestimates the dose in the lung because it disregards the lack of lateral electronic equilibrium and changes of scattered dose. The rebuild-up in the target observed with Monte-Carlo is ignored by Ray-Tracing. Behind the rebuild-up zone at the centre of the target, Ray-Tracing overestimates the dose because of the absence of scattering in lung tissue. On the edge of the target, in the PTV margin, the differences are even more pronounced because the lack of electronic equilibrium is dominant in this area, and Ray-tracing applies the change of density observed on the beam axis whereas the interface between lung and target is not at the same depth. The same kind of differences between every type A and type B algorithm can be observed: they are maximal in lung at the edge of the PTV (Figure 2). The smaller the lesion, and the beam, the higher the discrepancy between type A and type B algorithms in lung [12]. Because of that, modifying the prescription is more complicated for SBRT than for wide-field RT treating bigger volumes. Although the dose of 60 Gy is overestimated when calculated by a type A algorithm, local control has been very high [13], so this actual dose level should be maintained. Comparison between Pencil Beam and Collapsed Cone (Oncentra Masterplan, V4.1, Nucletron Elekta, Veenendaal, Netherlands) for the Varian Clinac® (Varian Medical Systems Inc., Palo Alto, CA) and comparison of Pencil-Beam and Monte-Carlo dose calculations for Novalis® (Brainlab AG, Feldkirchen, Germany) lead to the same conclusions. Current study will be applied to these treatment platforms.

A systematic review for NSCLC stage I SBRT was recently published [14] showing a wide variability in reporting. In the 45 identified studies there were 22 different treatment schemes. One dose level only, probably PTV based, was reported for each study, ranging from 15 Gy to 72.5 Gy in 1 to 12 fractions. In another paper van Baardwijk et al. try to determine the best therapeutic ratio [15] despite the differences between type A and B algorithms considering only dose at the edge of the PTV. The questions in these articles will not be answered correctly when considering only the PTV.

Our goal is to establish a consistent dose prescription and reporting in a heterogeneous environment that is applicable for different SBRT treatment modalities.

## Methods

A dose calculation experiment is proposed to discuss the different prescription methods. This exercise is not meant to be an exhaustive demonstration but should primarily provide a number of arguments to put into perspective the current prescription methods.

**Figure 3:**
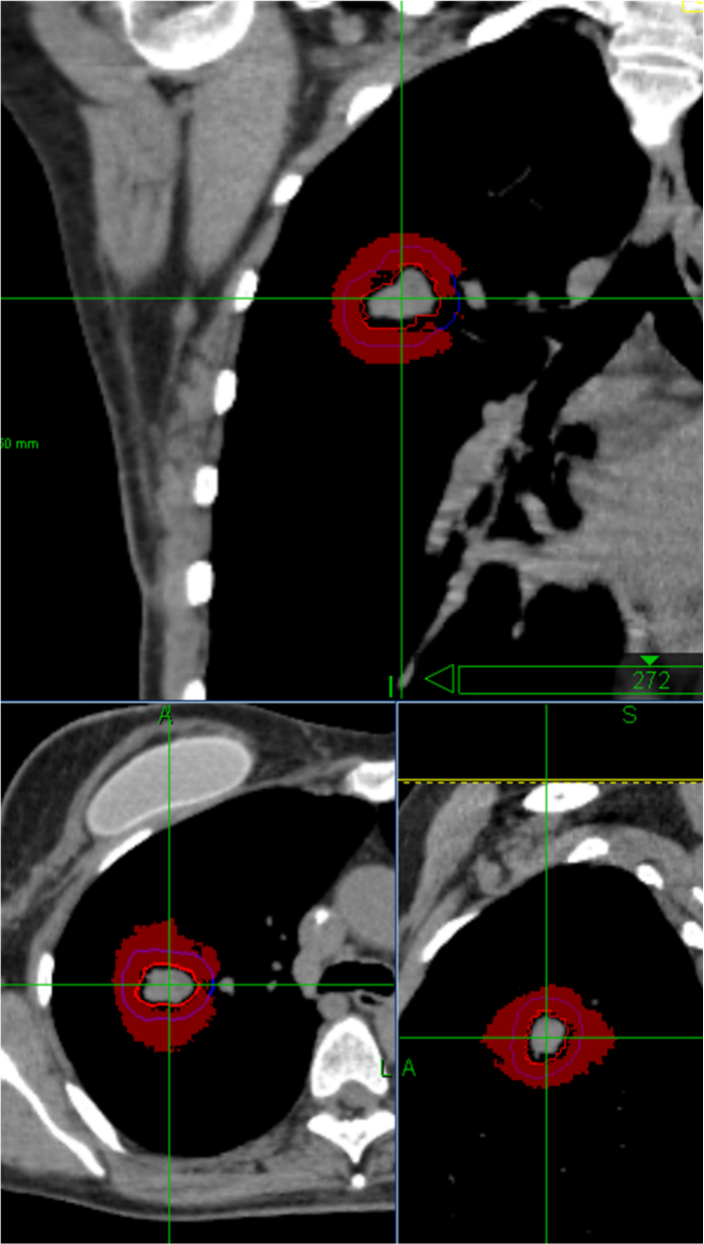
**Differences (in red) larger than 15 Gy for case 2 on Cyberknife between Ray-Tracing and Monte-Carlo.**

Three different cases with three different sizes of peripheral lung lesions (<1 cm, ~2 cm, ~4 cm) were examined (Figure 3). Each case was calculated with a type A and a type B algorithm on three different platforms: CyberKnife with Ray-Tracing and Monte-Carlo (Multiplan v.9.0, 1% statistical uncertainty and resolution of 1×1×1 mm), Novalis 6 MV with Pencil-Beam and Monte-Carlo (Iplan v.4.1.2, 0.5% statistical uncertainty and resolution of 2×2×1 mm), and Clinac 6 MV (Varian) with Pencil-Beam and Collapse Cone (Masterplan v4.0, resolution of 2×2×1 mm). The PTV was defined as GTV + 5 mm, for the treatment with gating or with real-time tracking, details of ballistics are given in Table 1. The targets were not enlarged for prophylactic treatment (RTOG 0236 & 0915).

**Table 1:** **Description of cases and irradiation techniques**

	**Case 1**	**Case 2**	**Case 3**
GTV	1.0 cm^3^	3.6 cm^3^	32.8 cm^3^
PTV	6.4 cm^3^	14.5 cm^3^	75.0 cm^3^
Novalis (Non-IMRT)	10 coplanar and non opposed beams	9 coplanar and non opposed beams	10 coplanar and non opposed beams
Cyberknife (circular collimators and flattening filter free)	62 non coplanar beams	58 non coplanar beams	52 non coplanar beams
Clinac (Non-IMRT) prescription like classical 3DRT with type A, extra-margin from PTV to field edge	5 coplanar and non opposed beams	7 coplanar and non opposed beams	7 coplanar and non opposed beams

Scenario A: 60 Gy was prescribed to 95% of the PTV using type A algorithms (RTOG 0236). For the Clinac plans, the margin from PTV to TV was 5 mm, as in conventional 3D-CRT, consequently the field sizes were larger than 3×3 cm^2^ for all cases. The GTV D98% (near min) and the D50% (median) were compared. Each plan was recalculated with a type B algorithm while keeping the monitor units per beam constant. The D95% of the PTV was calculated and again the D98% and D50% for the GTV were evaluated.

Scenario B: Because type A algorithms overestimate the delivered dose, 48 Gy instead of 60 was prescribed with type B algorithms, renormalizing the plans used in Scenario A, to 95% of the PTV, as proposed in the STARS protocol by the MD Anderson Cancer center. The different doses to the GTV were observed for all combinations of cases and platforms.

Scenario C: Finally, to avoid discrepancies among median GTV doses, a dose of 54 Gy was prescribed to this parameter using a type B algorithm. These plans were first optimized with type A algorithms to ensure a homogeneous photon fluence in the PTV (−5%, +15% of prescription dose), recalculated with type B algorithms and normalized in a way that the GTV D50% equals the prescription dose (i.e. 54Gy).

Because the three lesions were in the centre of lung, the doses to organs at risk – esophagus, ribs, and heart – were very low for all cases; only the normal tissue dose to lung was recorded. A large number of criteria concerning lung toxicity have been published [16]. Most of them were calculated for the current study, yet not all of them are published here for the sake of brevity. The lung volume receiving <10 Gy was presented to show that in all scenarios, the criteria proposed by AAPM report of TG 101 were fulfilled [17].

## Results

### Scenario A

When prescribing 60 Gy to 95% of the PTV using a type A algorithm, the maximum discrepancies in the median dose to the GTV were moderate among studied platforms: 3.6 Gy (6% of the prescription dose (Dp)), and cases: 4.5 Gy (7.5% of Dp) (Table 2). The maximum dose variance among the nine plans was 6 Gy (10% of Dp), which is common in SBRT in homogenous tissues. For the GTV near-minimum dose (D98%), used as proposed by the ICRU report 83, the maximum differences were comparable: 4 Gy (6.7% Dp) among techniques and 2.4 Gy (4% Dp) among cases. For the two platforms with conventional stereotactic margins, Novalis and CyberKnife, higher median doses were obtained for larger lesions. For the Clinac using a 5-mm margin from PTV to TV, i.e. using a conventional prescription as for classical 3DRT, the median dose was almost the same for the three cases.

**Table 2:** **Prescription and calculation with type A algorithms (scenario A)**

**PTV D95% = 60 Gy type A**									
	**GTV D98% type A (Gy)**			**GTV D50% type A (Gy)**			**Normal lung receiving less than 10 Gy (cm** ^**3**^ **)**		
	**Case 1**	**Case 2**	**Case 3**	**Case 1**	**Case 2**	**Case 3**	**Case 1**	**Case 2**	**Case 3**
Novalis	61.5	62.0	61.8	61.6	63.2	64.1	3808	3468	2227
Cyberknife	61.6	64.0	62.3	62.8	64.9	67.3	3799	3488	1992
Clinac	60.7	60.0	60.3	62.3	61.3	64.0	3681	3308	1804

### Prescription with type A algorithms and re-calculation with type B algorithms

**Figure 4:**
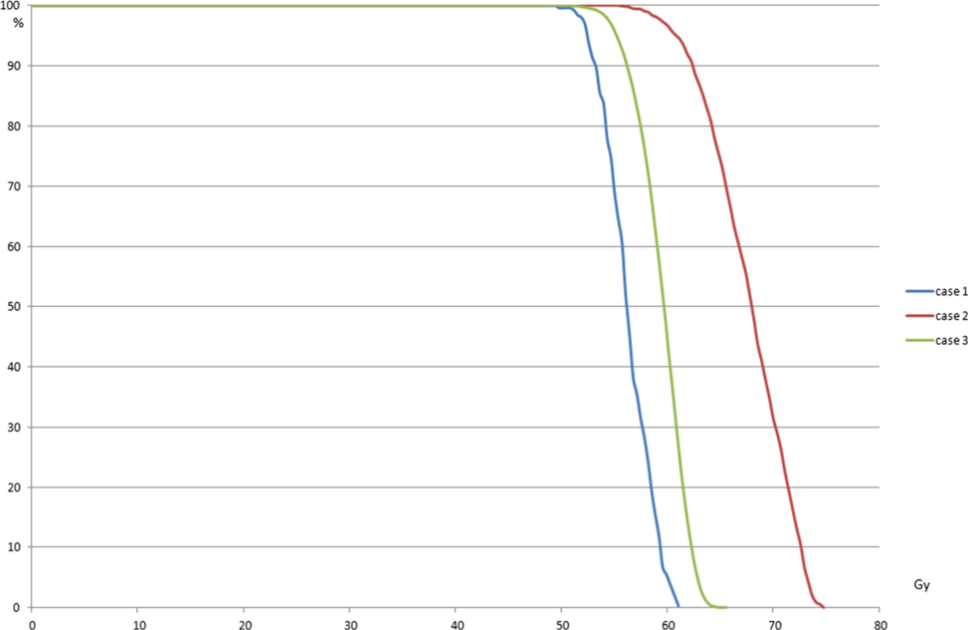
**Case 2: GTV DVH for PTV based prescription for the 3 different platforms (type B algorithms).**

When a type B algorithm was used, for each platform, the PTV D95% was smaller than the prescription dose according to the type A algorithm (Figure 4). The maximum difference was 19.6 Gy (32.7% Dp) (Table 3). Moreover a large range of doses (40.4–54.4, 23.3% Dp) was observed. For the GTV D98%, the maximum difference between cases was 12.4 Gy (20.7% Dp), the maximum difference between platforms was 3.6 Gy (6% Dp). For the GTV D50%, the maximum difference between cases was 13.2 Gy (22%) and the maximum difference between platforms was 3.2 (5.3%). The differences regarding the volume of lung that received less than 10 Gy for cases 1 and 2 (3% maximum) were small. Type A algorithms overestimated the high dose but the low doses (<10 Gy) are quite similar.

**Table 3:** **Calculation with type B algorithms with type A algorithms prescription (scenario A, same monitor units)**

**PTV D95% = 60 Gy type A**												
	**PTV D95% type B (Gy)**			**GTV D98% type B (Gy)**			**GTV D50% type B (Gy)**			**Normal lung receiving less than 10 Gy (cm** ^**3**^ **)**		
	**Case 1**	**Case 2**	**Case 3**	**Case 1**	**Case 2**	**Case 3**	**Case 1**	**Case 2**	**Case 3**	**Case 1**	**Case 2**	**Case 3**
Novalis	43.7	40.5	52.2	47	47.7	59.4	49.8	53.9	63	3858	3456	2189
Cyberknife	42.7	40.4	49.5	46.3	49.7	55.9	50	57.1	61.5	3824	3537	2090
Clinac	44.6	50.3	54.4	48.7	51.3	58.1	51.4	57	62.1	3757	3259	1817

### Scenario B

**Figure 5:**
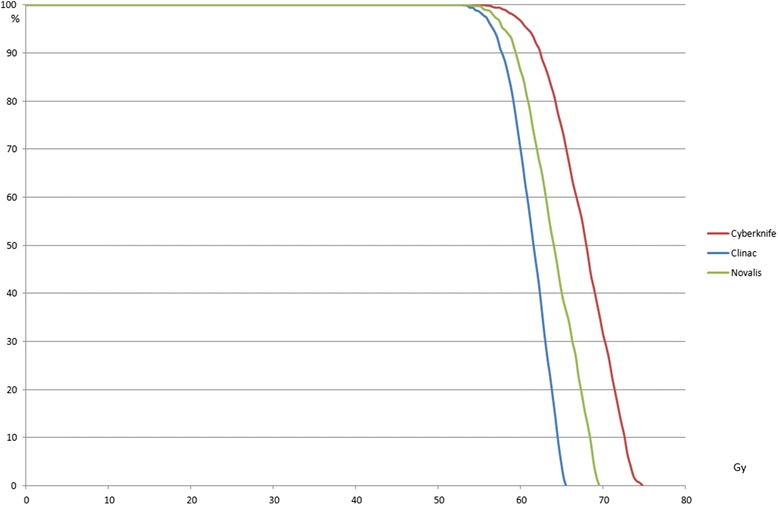
**Cyberknife: GTV DVH for PTV based prescription for the 3 cases calculated with Monte-Carlo.**

With a prescription of 3x16 Gy to 95% of the PTV using type B algorithms, the maximum differences for median doses of the GTV were: 13.4 Gy (28% of the prescription dose) among the platforms and 11.6 Gy (24.2%) among cases (Table 4). For GTV D98% (near-min) the maximum differences were comparable: 7 Gy (14.6%) (Figure 4) among platforms and 10.1 (21%) among cases (Figure 5).

**Table 4:** **Calculation with type B algorithms with prescription on PTV (scenario B)**

**PTV D95% = 48 Gy type B**									
	**GTV D98% type B (Gy)**			**GTV D50% type B (Gy)**			**Normal lung receiving less than 10 Gy (cm** ^**3**^ **)**		
	**Case 1**	**Case 2**	**Case 3**	**Case 1**	**Case 2**	**Case 3**	**Case 1**	**Case 2**	**Case 3**
Novalis	51.6	56.5	54.6	54.7	63.9	57.9	3824	3352	2219
Cyberknife	52.0	59.0	54.2	56.2	67.8	59.6	3777	3446	2218
Clinac	52.4	49.0	51.3	55.3	54.4	54.8	3723	3294	1927

The reader is kindly referred to the supporting material for more details and additional DVHs (Additional file 1).

### Scenario C

As illustrated above (scenario A), for plans prescribed at 95% of the PTV using type A algorithms, a large range of GTV50% values was obtained when recalculated using type B algorithms, Therefore we suggested a prescription dose of 54 Gy to 50% of the GTV when using a type B algorithm.

The maximum differences for GTV D98% were 1.8 Gy (3.4% of the prescription dose) among the platforms and 3.1 Gy (5.8%) among cases (Table 5) Of course with this scenario there are differences for the PTV D95%, the maximum is 9.4 Gy (17% of the prescription dose) among the platforms and 5.3 Gy (9.7% of the prescription dose) among cases.

**Table 5:** **Calculation with type B algorithms with prescription on GTV D50% (scenario C)**

**GTV D50% = 54 Gy**									
	**GTV D98% type B (Gy)**			**PTV D95% type B**			**Normal lung receiving less than 10 Gy (cm** ^**3**^ **)**		
	**Case 1**	**Case 2**	**Case 3**	**Case 1**	**Case 2**	**Case 3**	**Case 1**	**Case 2**	**Case 3**
Novalis	51.0	47.8	50.9	47.4	40.6	44.7	3829	3455	2284
Cyberknife	50.0	47.0	49.1	46.1	38.2	43.5	3794	3563	2309
Clinac	51.2	48.6	50.5	46.9	47.7	47.3	3735	3299	1945

## Discussion

The accuracy of Monte Carlo dose calculation for the studied platforms, Novalis and CyberKnife, has been demonstrated [18,19] using anthropomorphic phantoms [20]. Therefore, it is advisable that advanced algorithms are used to obtain more accurate dose distributions to targets and organs at risk. The question is how to apply the well established prescription protocols that lead to positive clinical results.

On one hand, there are a lot of studies that report excellent local control [11,21,22] with different prescription schemes in which only the prescription dose to the PTV is provided without mentioning the algorithm used. On the other hand, differences between type A and type B algorithms have been observed in many studies [4,5,23,24] without clear consequences regarding the prescription. Hurkmans et al. have proposed for the ROSEL study [25] different dose conformity requirements according to the size of the PTV and the type of the algorithm used. The prescription dose was the same for all lesions based on the PTV, but the heterogeneity in the PTV and the dose constraints for the lung varied according to the size of the PTV. Van der Voort van Zyp et al. [26] have also performed a retrospective analysis of Monte-Carlo calculations on cases treated in their institution. They proposed three dose levels based on the size of the tumor calculated with Monte-Carlo and prescribed to the 95% of the PTV (48 Gy for tumors <3 cm, 51 Gy for tumors of 3–5 cm, and 54 Gy for tumors >5 cm), which can be considered as the most consistent proposition to date.

For PTV based prescription using type A algorithms a small variability for GTV D98% and GTV D50% among cases, and among platforms was obtained. Recalculation of the plans using a type B algorithm shows that, there is a dose escalation according to the size of the GTV with few discrepancies between the platforms (3.2 Gy, 5.2% of the prescription dose). When a type A algorithm is used with a PTV based prescription the dose delivered to the GTV is higher for larger lesions. That may explain the good local control of all the studies performed using type A algorithms. This result, also described in detail by van der Voort van Zyp et al. [26], led to their proposition of the three dose levels.

As the PTV includes a low-density region, lacking electronic equilibrium, the prescription, using type B algorithms, should not be based on this volume. For example, the prescription of 48 Gy to the PTV D95% in case 2 leads to a higher GTV median dose than for case 3 which is a larger lesion. Even if we prescribed like van der Voort van Zyp et al. (18) [26] suggest, i.e., 48 Gy to 95% of the PTV for case 2 and 51 Gy for case 3, the GTV median dose remains inferior to that of case 2 (61.5 Gy vs. 63 Gy for Novalis, 63.3 Gy vs. 67.8 Gy for CyberKnife). If the same level of dose should be delivered to larger lesions, a PTV-based prescription should not be used.

The PTV is a fictitious volume created to ensure that the absorbed dose to the target equals the prescription dose, taking into account positioning uncertainties. Because of the low density surrounding the GTV, the minimum dose to the PTV is calculated in a low density region whereas the GTV has a density close to 1.

**Figure 6:**
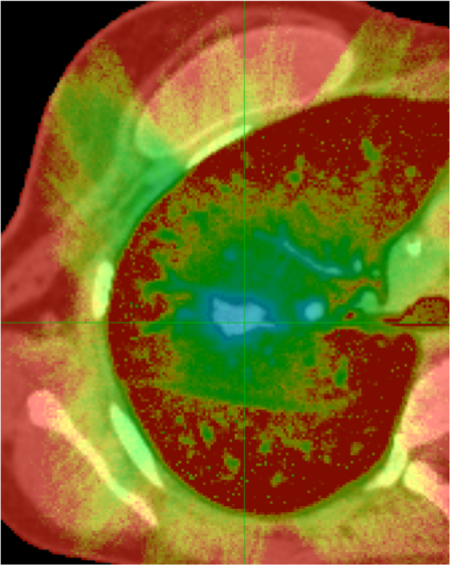
**Map of % of statistical uncertainties for Monte-Carlo-Calculation for case 2 and Cyberknife.**

The PTV D95% depends on the lung density around the lesion and does not predict the dose to the GTV (28% variability in the median prescription dose and 14.6% for Dnear-min for the different treatment devices in our exercise). The difference between PTV D95% and GTV dose depends on the size of the lesion, lung density, location, treatment platform and even the operator. Our exercise is not intended to be exhaustive but only to show that one PTV criterion is not enough to characterize the dose distribution. The PTV margin in lung can be considered as the “flash” region margin used for tangential breast field, where part of the PTV is in air, while it is not used for prescription. Furthermore the statistical uncertainty of Monte-Carlo is up to 5% higher (or more for extreme case of low density lung) in the PTV periphery than on the GTV median dose (Figure 6) because there are fewer histories in low density region. With a prescription based on the PTV, the discrepancies between treatment systems are much more substantial with type B than with type A algorithms; 28% vs. 6%.

The best way to avoid these discrepancies among cases and treatment platforms is to prescribe to the GTV median dose. The GTV median dose is the most representative dose, in that the majority of the lesion receives ±5% of the median dose even in the example with the highest degree of heterogeneity: case 2 treated with the CyberKnife. We believe that the GTV D50% is more relevant than the GTV near-minimum (D98%) because the GTV encompasses sometimes small low density regions, lacking electronic equilibrium too, so the uncertainty is larger than for the GTV D50%. However we think we should report this value.

**Figure 7:**
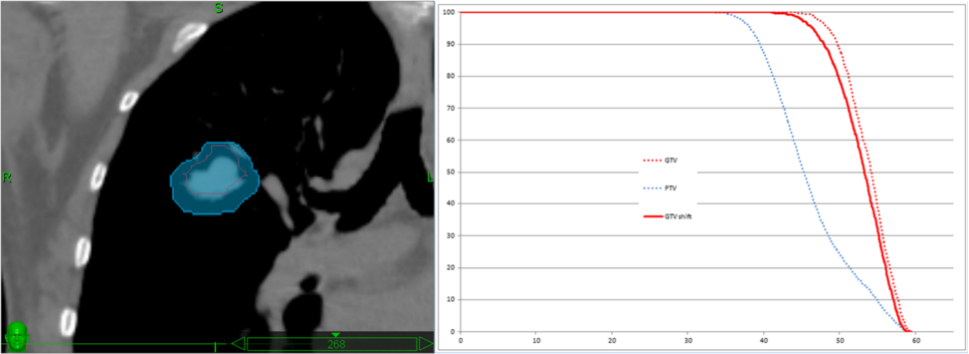
**Effect of a shift of the GTV in the envelop of the PTV for case 2 and Cyberknife: The red contour indicates the position of the GTV shifted within the PTV (blue).**

During plan optimization the PTV needs to be targeted entirely though, because the GTV can be anywhere inside the PTV. This task is best achieved using a type A algorithm, available in all TPSs. In clinical practice, like in the exercise described in current article, a plan is created using a type A algorithm, covering 95% of the PTV by the prescription isodose. A steep gradient around the PTV is not really needed, because the effect on the DVH of the lung is very small and because the CTV extensions are not known, so a relatively homogenous fluence in the target is maintained. Then a type B algorithm is used to recalculate and rescale the result and to prescribe the dose to the GTV median dose (GTV D50%). As the median does not really depend on the minimum, the GTV median dose is almost constant no matter where the GTV is within the PTV envelop and, consequently, is very representative of the dose delivered and thus the biological impact to the tumor. For example if for case 2 treated with the Cyberknife, where the dose heterogeneity is largest (difference between PTV D95% and GTV50% and GTV98%), the GTV is shifted 5 mm towards the dose minimum in the PTV (overriding density of PTV-GTV and GTV shifted), (see Figure 7), the median dose to the GTV is 53.2 Gy instead of 54 Gy (a 1.5% variation): because of the rebuild-up effect the isodose moves with the GTV. For all other studied situations the variation would be lower because the dose heterogeneity is lower within the PTV. The combination of optimizing to the PTV using a type A algorithm, and prescribing to the median GTV dose using a type B algorithm is thus a very robust method.

The reoptimization with type B algorithms, studied in this article, is not described because it increases uncertainties of delivered dose. To compensate the lack of electronic equilibrium the system will increase the fluence in this region, this results in a non robust plan, as the GTV will be overexposed when it moves into the regions with increased photon fluence. As in all situations of lack of electronic equilibrium, like superficial neck nodes in build-up region, optimization increases the uncertainty on the delivered dose. New algorithms taking into account 4DCT and uncertainty of patient position should be used to have a robust optimization. As this robust optimization algorithms are not yet available in our TPSs, the method proposed in current work (optimizing on the PTV using a type A algorithm followed by recalculation using a type B algorithm) provides the best available alternative for the moment.

In this experiment, the breathing motion was considered controlled. If the PTV is defined from an ITV which includes all the positions of the GTV during the breathing cycle, the beam sizes used were larger so the lack of lateral electronic equilibrium was smaller but, still, the PTV D95% did not predict the minimum dose to the GTV, because the PTV margin consisted mostly of a low-density region. Therefore, also for the ITV method, we think it is better to prescribe to the GTV median. Without any gating or real-time tracking, the dose calculation is only a snapshot, but even then prescribing to the GTV D50% is more precise because it takes into account the rebuild-up. Actually the larger the margin the larger the difference between PTV minimum and GTV dose. That means that a large uncertainty in GTV position leads to a higher delivered dose. A GTV based prescription avoids this drawback.

## Conclusion

The right dose needed for lung lesions treated by SBRT is not yet known. Advanced algorithms are needed and the GTV dose should be reported because the PTV prescription does not predict the dose to the GTV. Every team should report GTV D50% and D98% to enable a comparison of all results.

We suggest using type A algorithms to target the PTV during optimization and to recalculate dose with type B algorithms rescaling the prescription to the GTV median dose. Until robust optimization algorithms will be introduced in all TPSs, this appears to be the most robust way to avoid discrepancies from device to device and case to case and this with or without motion management (tracking, gating).

## Additional file

Additional file 1
**Slideshow.**
