# Supplementary material for: GTV-based prescription in SBRT for lung lesions using advanced dose calculation algorithms
Source: Radiat Oncol. 2014 Oct 16;9:223. doi: 10.1186/s13014-014-0223-5 (PMC4205279; doi:10.1186/s13014-014-0223-5)
Supplement: Additional file 1 — Slideshow. [file 13014_2014_223_MOESM1_ESM.pdf]

# GTV-BASED PRESCRIPTION IN SBRT FOR LUNG LESIONS USING ADVANCED DOSE CALCULATION ALGORITHMS

**Thomas Lacornerie*, Albert Lisbona†, Xavier Mirabelʃ, Eric Lartigauʃ, Nick Reynaert***

*Service de Physique Médicale, Centre Oscar Lambret, Lille, France
†Service de Physique Médicale, Institut de Cancérologie de l'Ouest, Nantes, France
ʃDépartement Universitaire de Radiothérapie, Centre Oscar Lambret, Lille, France

# Exercise

- 3 cases with lesions of different sizes
  - <1 cm , ~2 cm, ~4 cm
- 3 different platforms (linac & TPS), for every case dose calculation using type A algorithm and type B algorithm :
  - Clinac (Pencil Beam et Collapsed Cone)

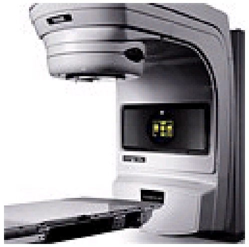

  - Novalis (Pencil Beam et Monte-Carlo)

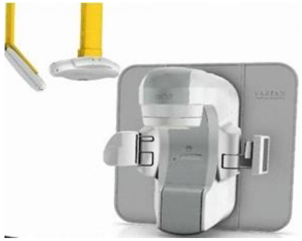

  - Cyberknife (Ray-Tracing et Monte-Carlo)

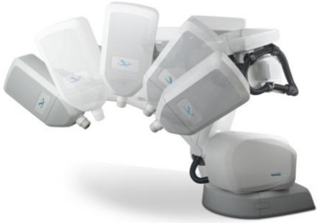

UNICANCER
Centre Oscar Lambret
Centre Régional de Lutte contre le Cancer

PTV (=GTV+5mm) D95% = 48 Gy

UNICANCER

Centre Oscar Lambret
Centre Régional de Lutte contre le Cancer

# Case 1 lesion < 1 cm

Clinac

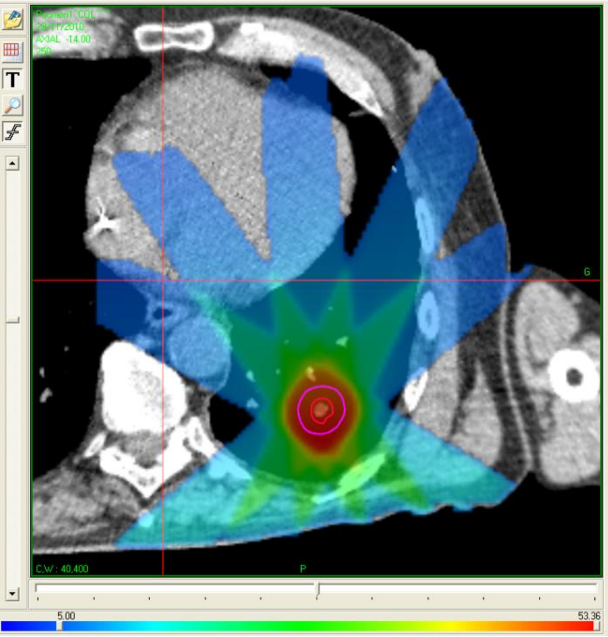


Novalis

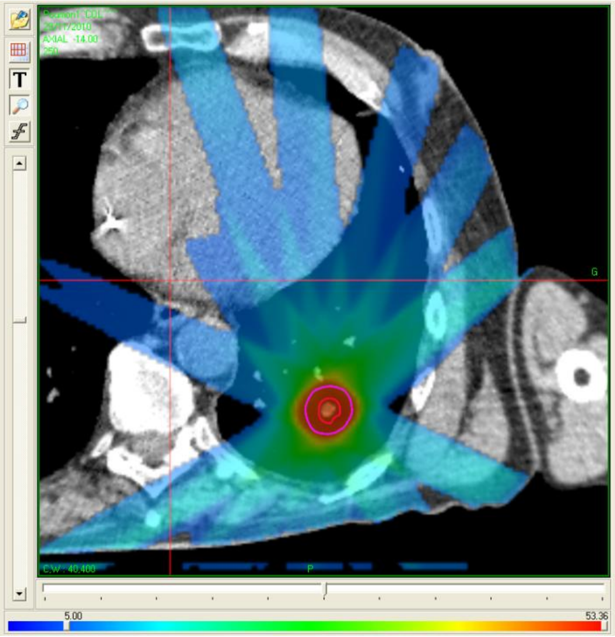


Cyberknife

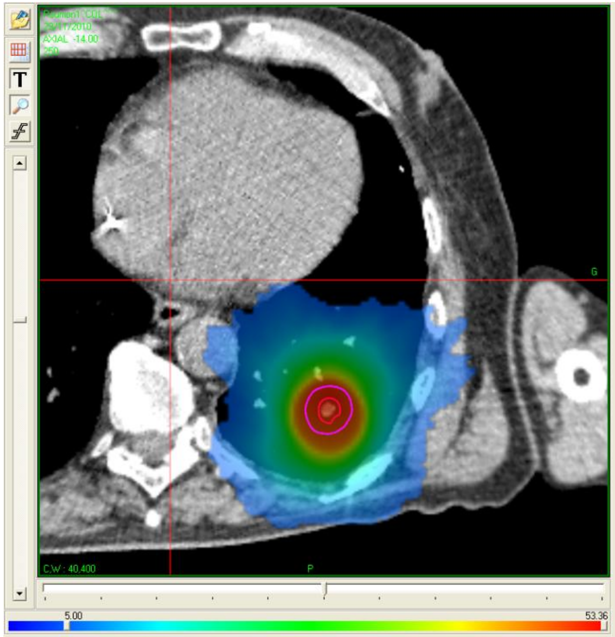


type A algorithms

doses > 5 Gy

# Case 1 lesion < 1 cm

Clinac

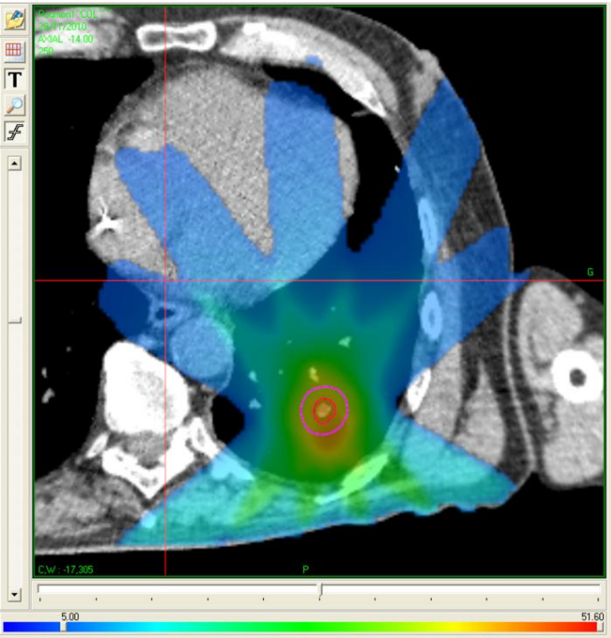


Novalis

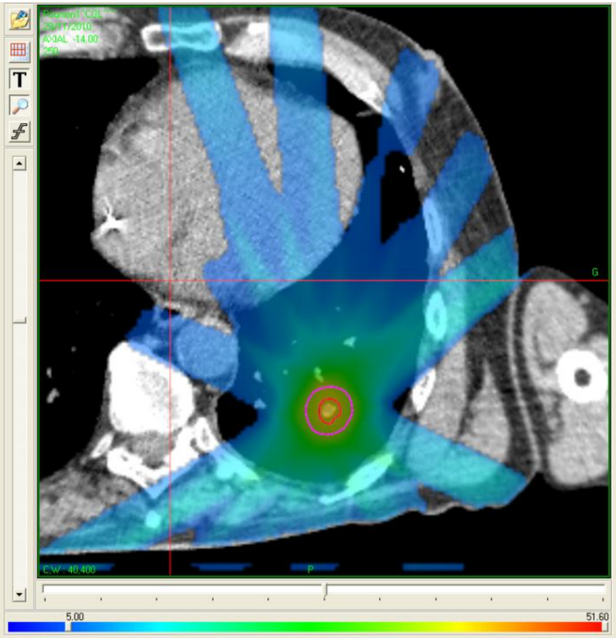


Cyberknife

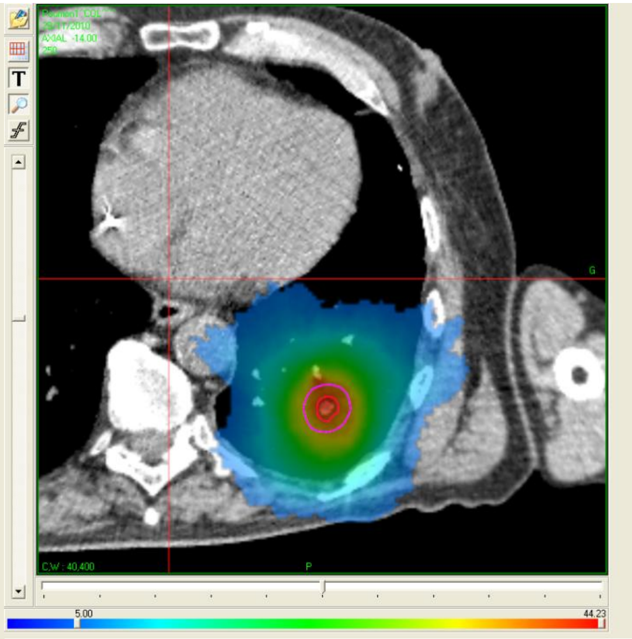


type B algorithms

same plans i.e. same m.u./beam

doses > 5 Gy

# Case 1 lesion < 1 cm

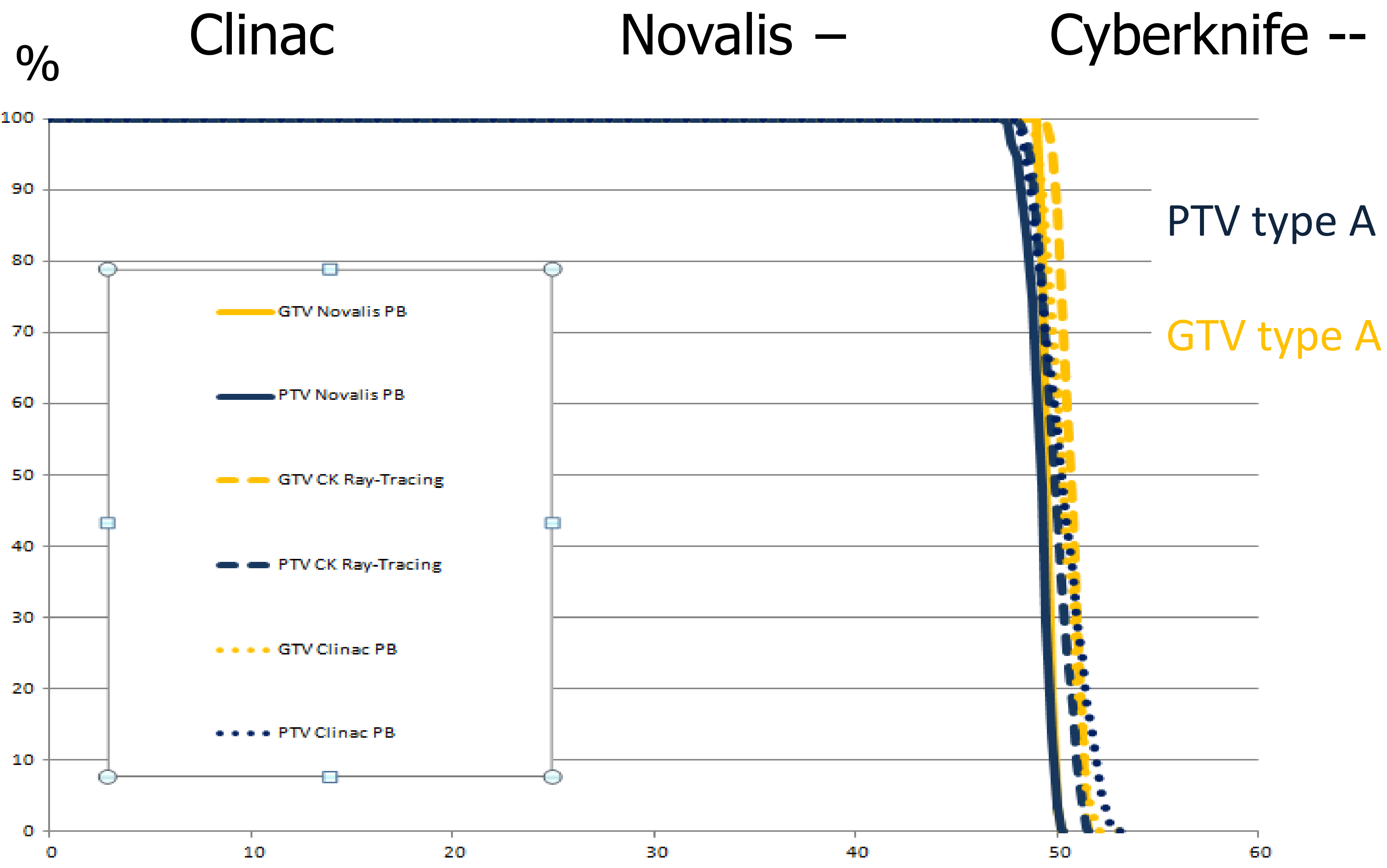

# Case 1 lesion < 1 cm

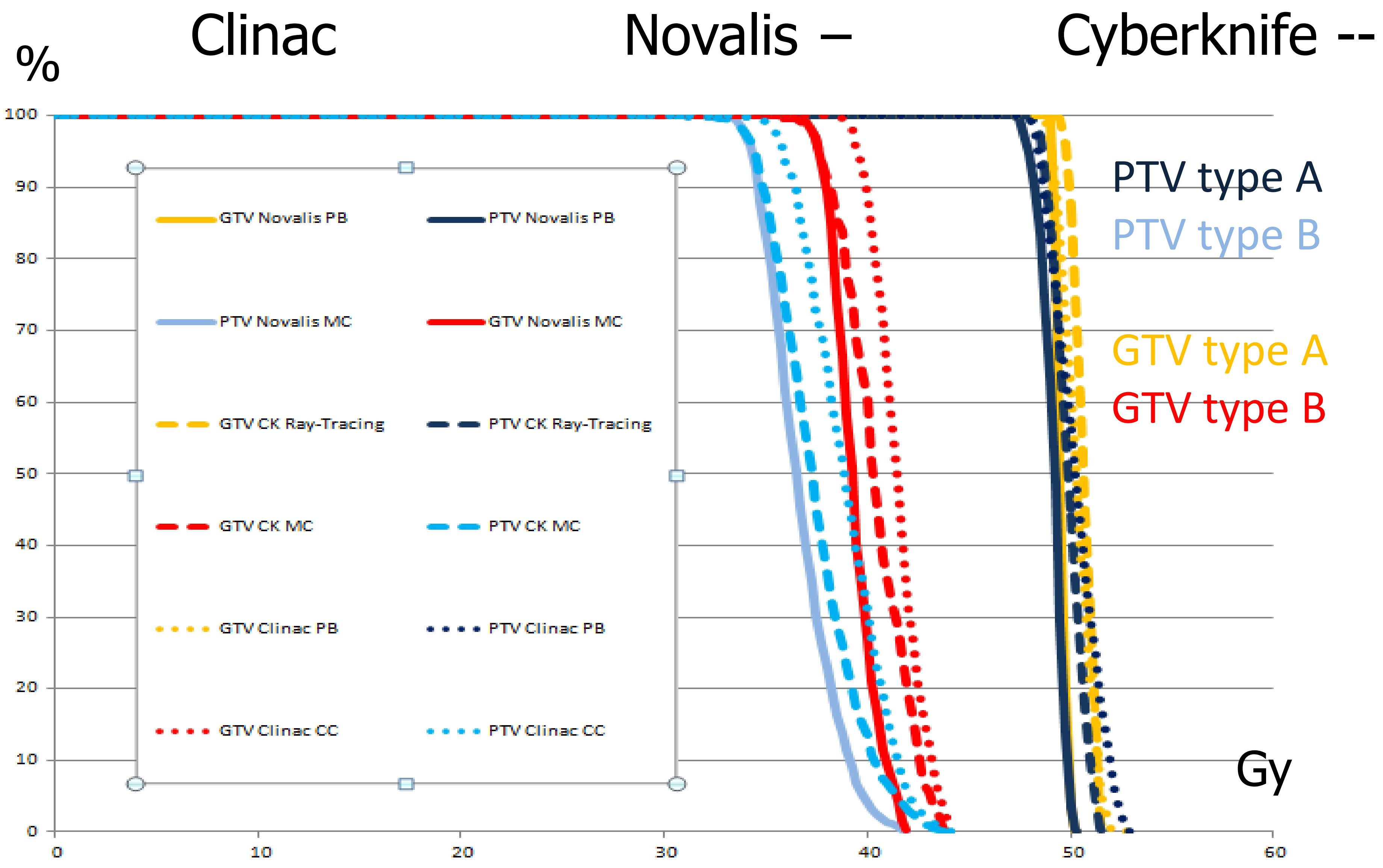

UNICANCER Centre Oscar Lambret Centre Régional de Lutte contre le Cancer

# Algorithms type A & B

- ✓ Type A algorithms overestimate dose because they don't take into account modification of lateral electron transport

- ✓ We have to use type B algorithms or Monte-Carlo which have demonstrated their consistency with measurements

UNICANCER Centre Oscar Lambret Centre Régional de Lutte contre le Cancer

- ✓ Proceed the exercise prescribing 48 Gy to 95% of PTV (=GTV+5 mm) using type B algorithms

# Case 2 (lesion ~2cm)

Novalis

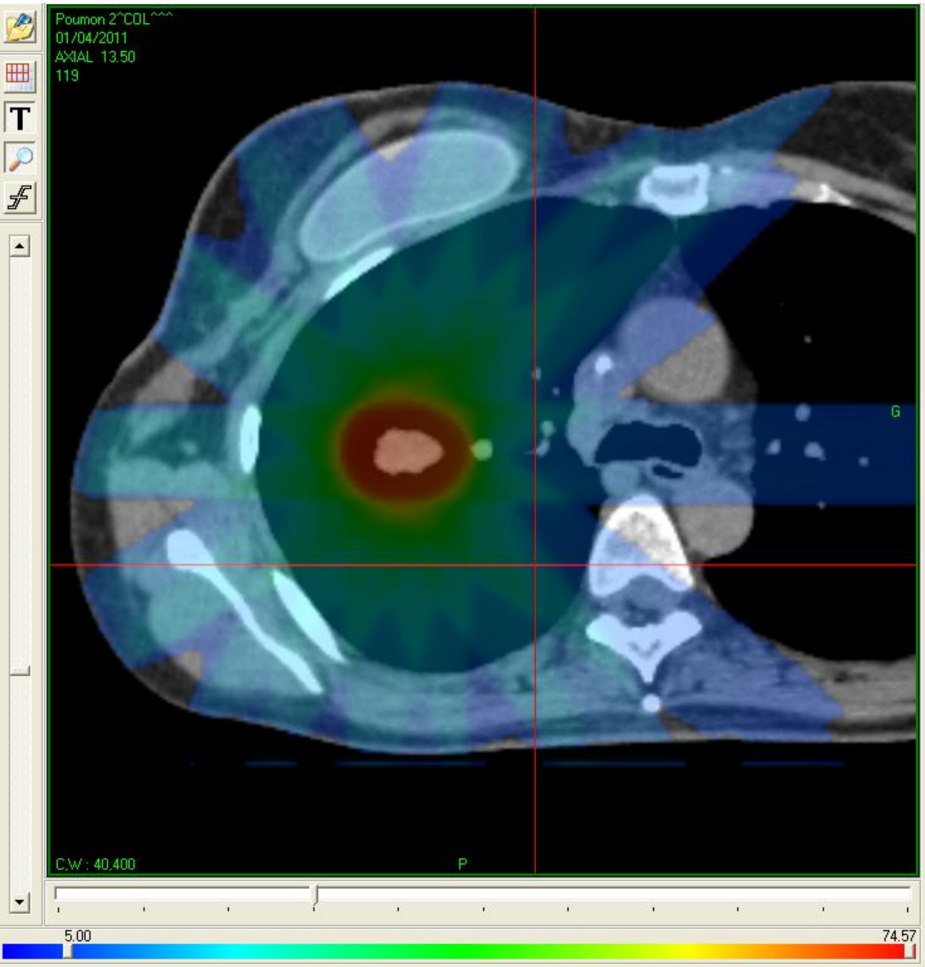


Cyberknife

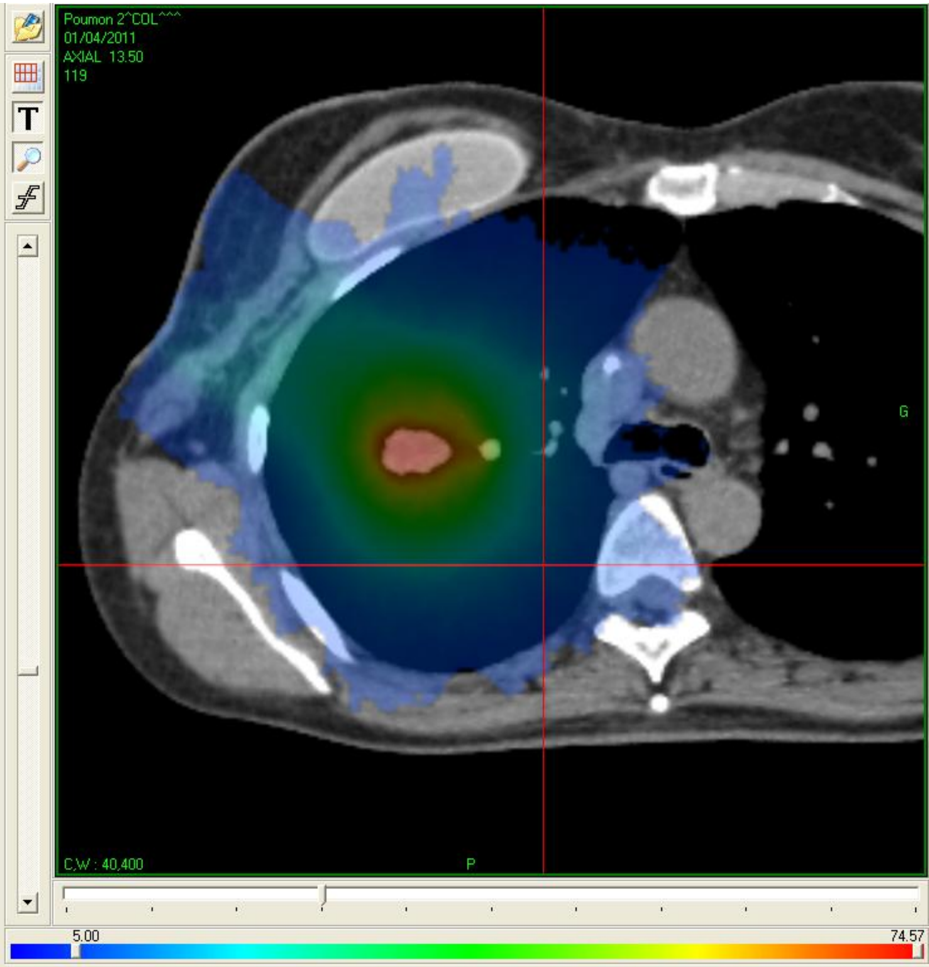

# Case 3 (lesion ~ 4 cm)

Novalis

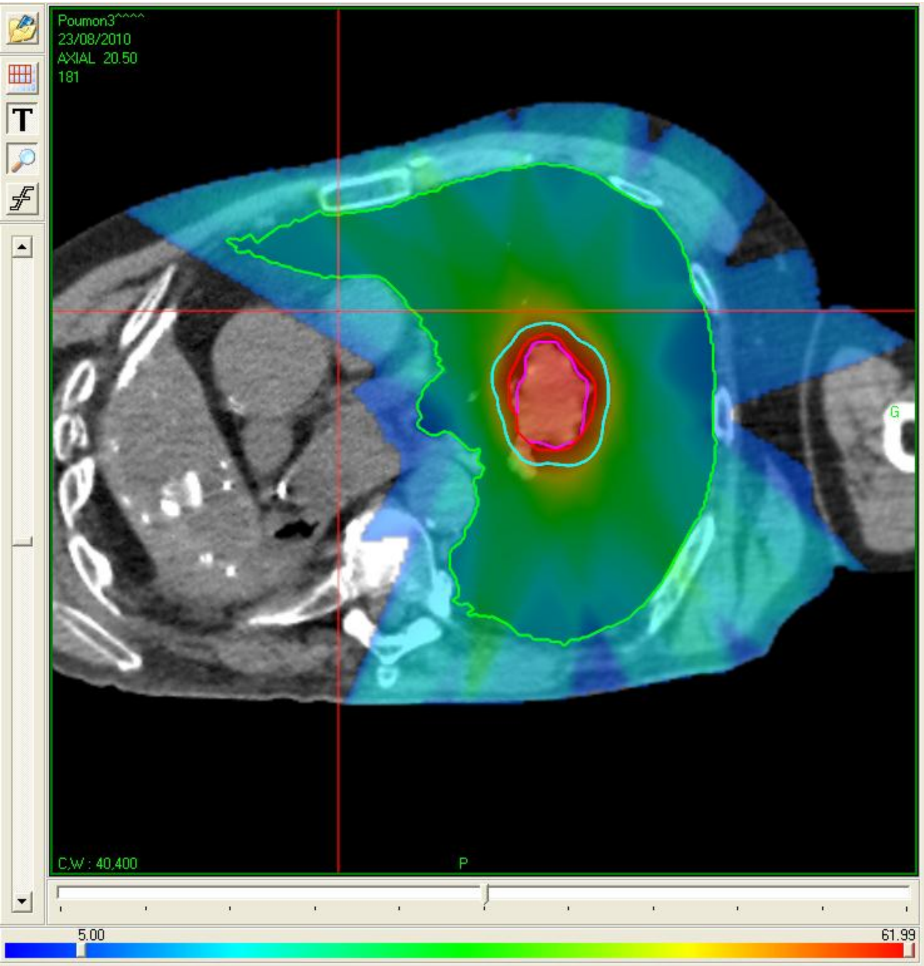


Cyberknife

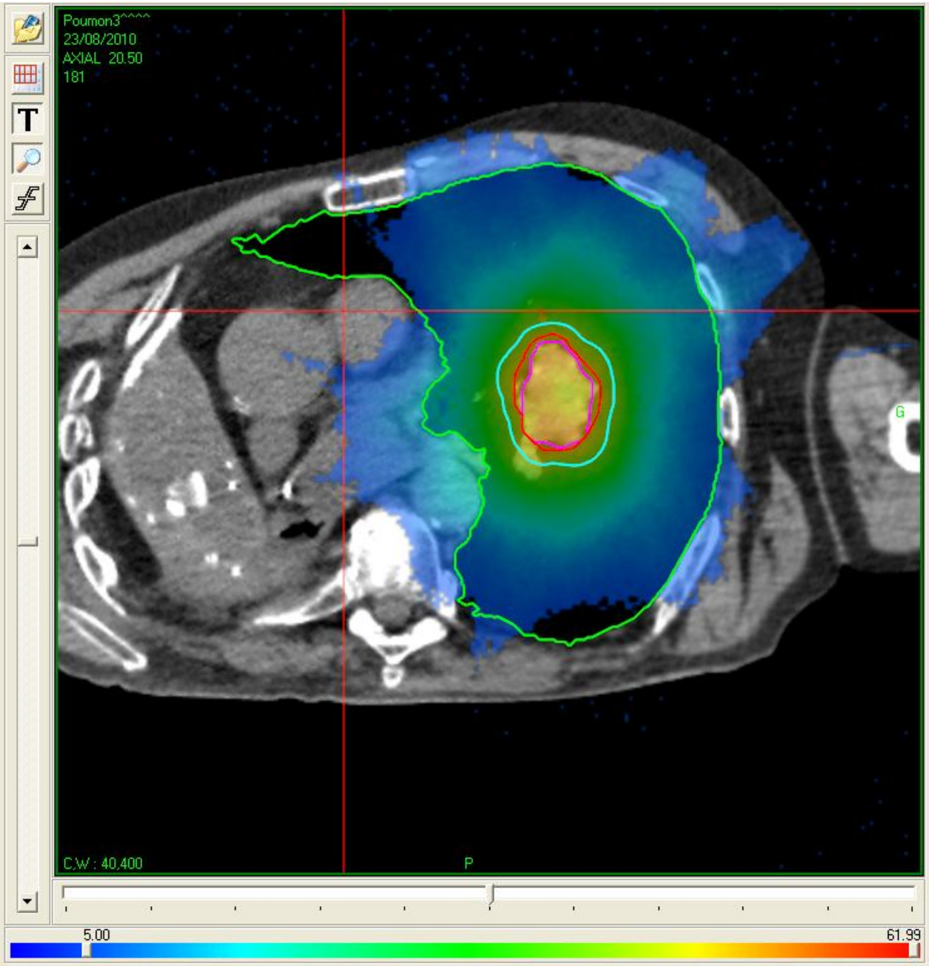

# Case 1

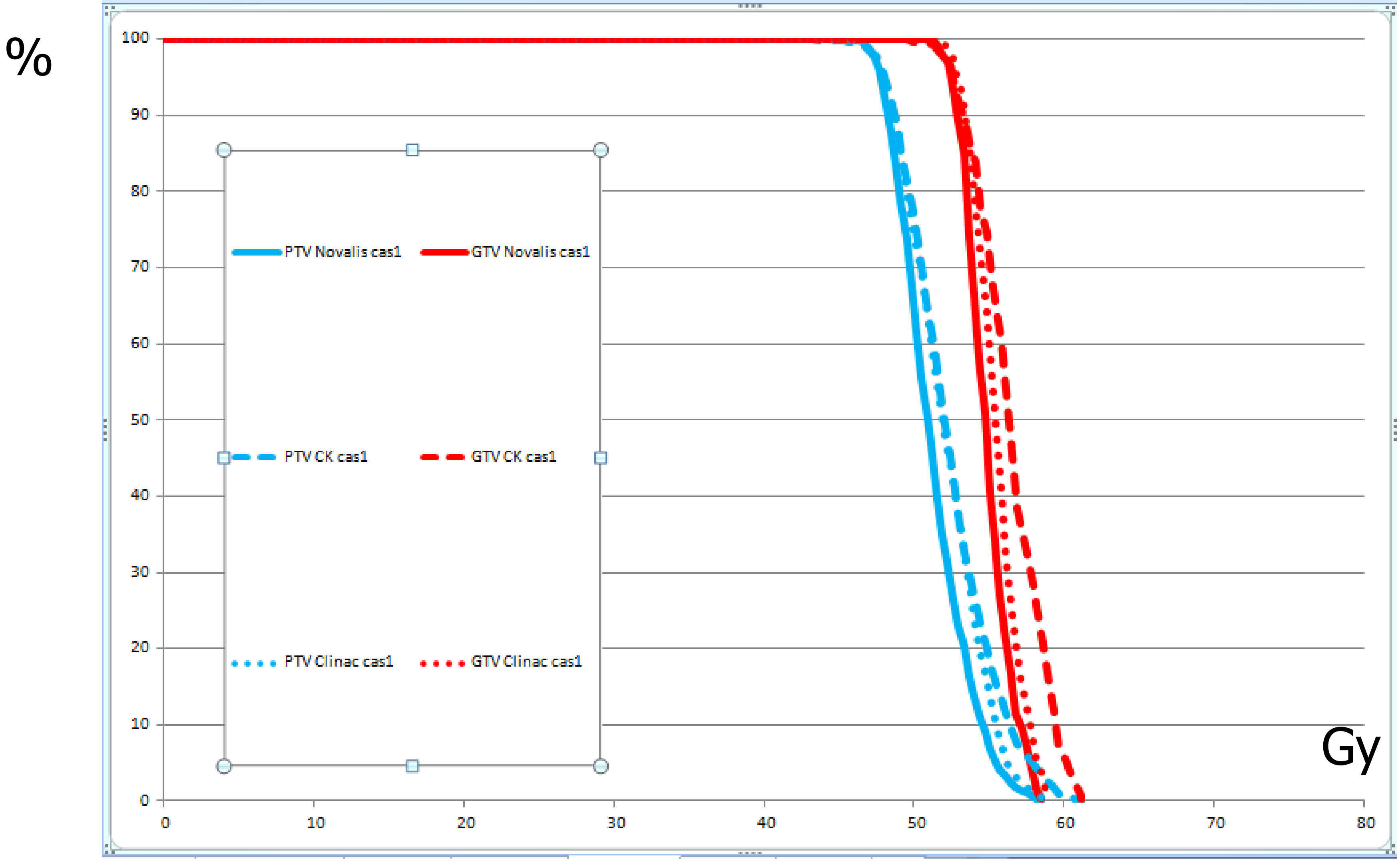
%
100
90
80
70
60
50
40
30
20
10
0
PTV Novalis cas1
GTV Novalis cas1
PTV CK cas1
GTV CK cas1
PTV Clinac cas1
GTV Clinac cas1
0
10
20
30
40
50
60
70
80
Gy

UNICANCER Centre Oscar Lambret Centre Régional de Lutte contre le Cancer

# Case 1 & 3

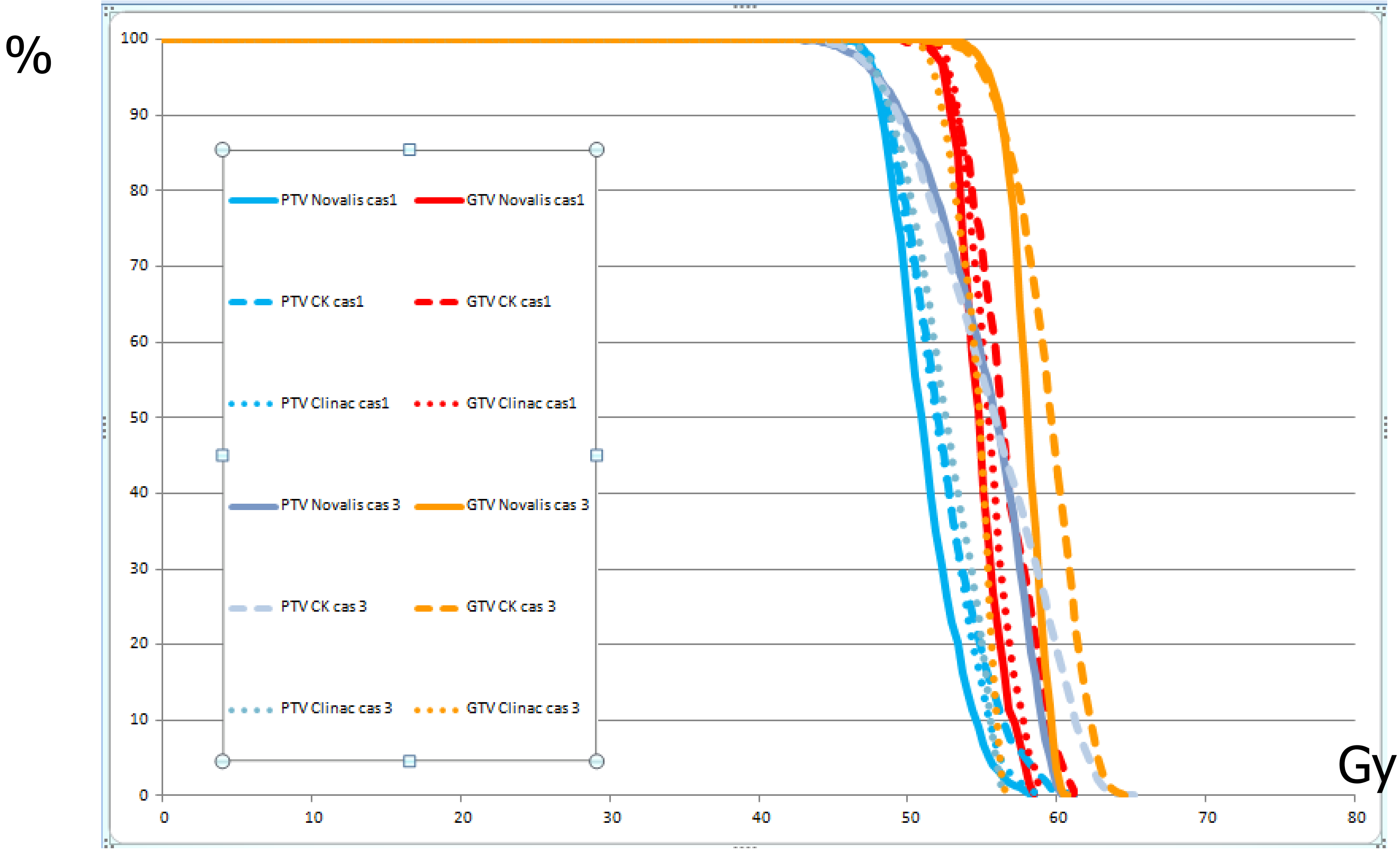

# Case 1, 2 & 3

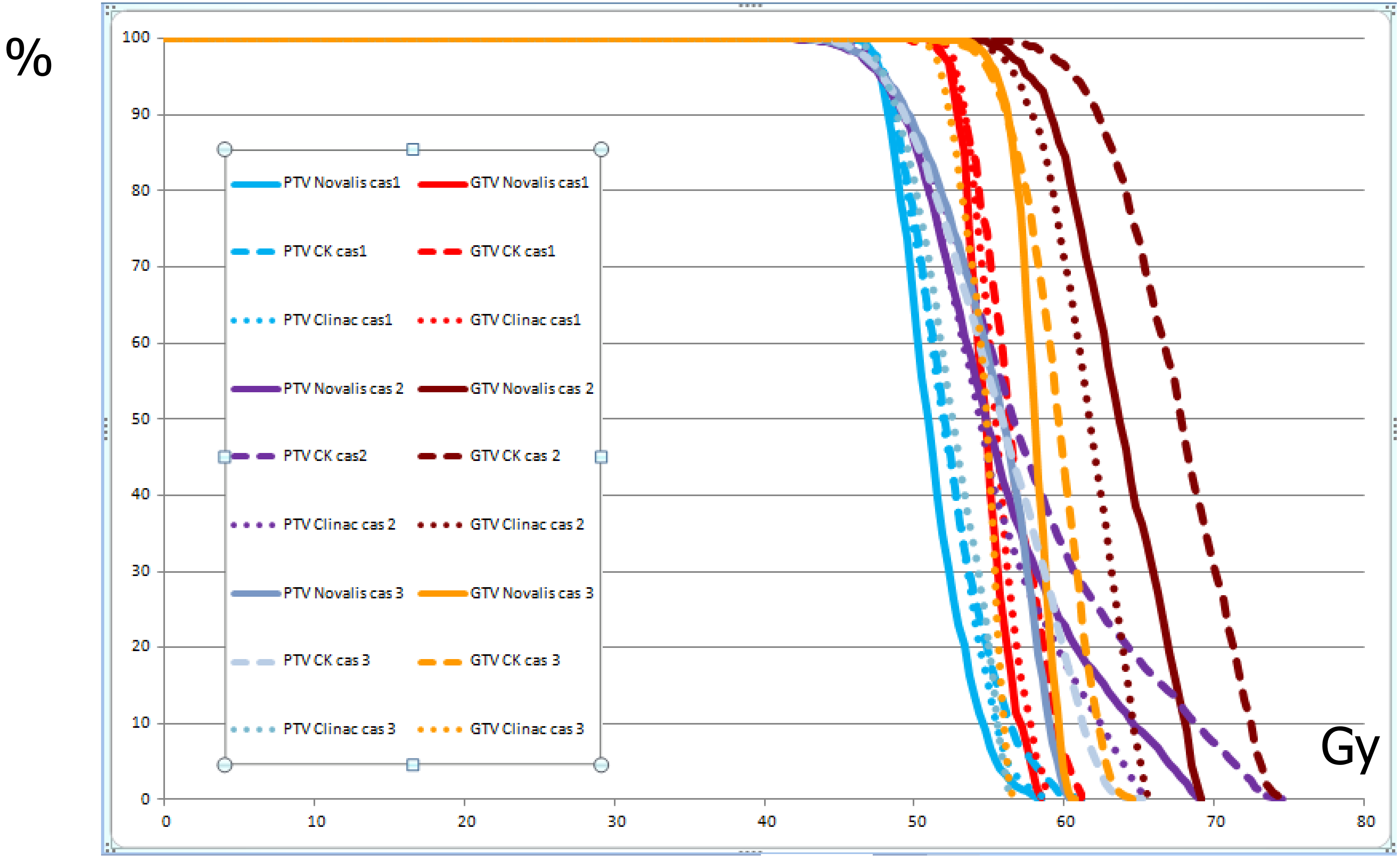

%
100
90
80
70
60
50
40
30
20
10
0
0
10
20
30
40
50
60
70
80
Gy
PTV Novalis cas1
GTV Novalis cas1
PTV CK cas1
GTV CK cas1
PTV Clinac cas1
GTV Clinac cas1
PTV Novalis cas 2
GTV Novalis cas 2
PTV CK cas2
GTV CK cas 2
PTV Clinac cas 2
GTV Clinac cas 2
PTV Novalis cas 3
GTV Novalis cas 3
PTV CK cas 3
GTV CK cas 3
PTV Clinac cas 3
GTV Clinac cas 3

# Case 1, 2 & 3

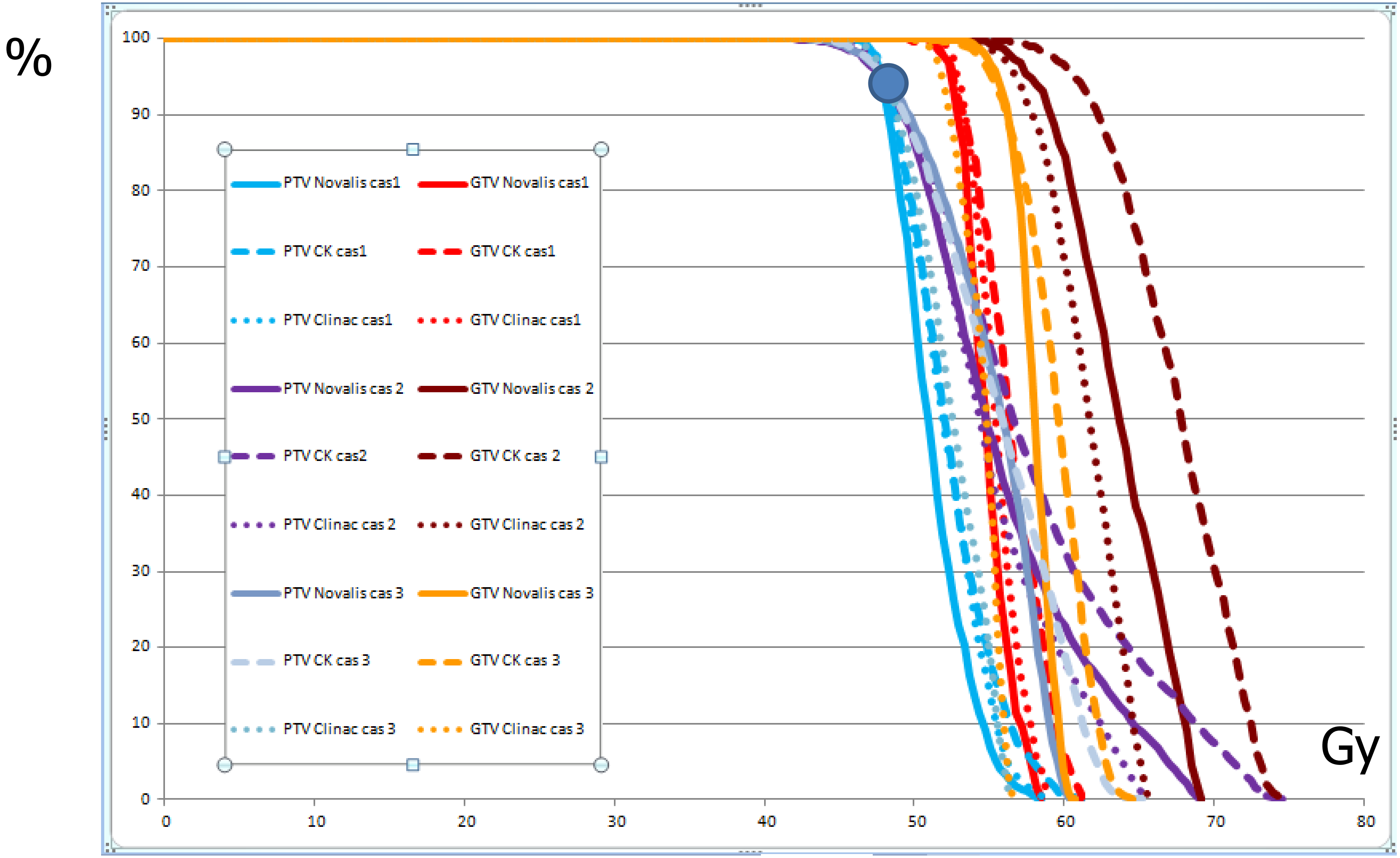

%
100
90
80
70
60
50
40
30
20
10
0
0
10
20
30
40
50
60
70
80
Gy
PTV Novalis cas1
GTV Novalis cas1
PTV CK cas1
GTV CK cas1
PTV Clinac cas1
GTV Clinac cas1
PTV Novalis cas 2
GTV Novalis cas 2
PTV CK cas2
GTV CK cas 2
PTV Clinac cas 2
GTV Clinac cas 2
PTV Novalis cas 3
GTV Novalis cas 3
PTV CK cas 3
GTV CK cas 3
PTV Clinac cas 3
GTV Clinac cas 3

# Case 1, 2 & 3

%

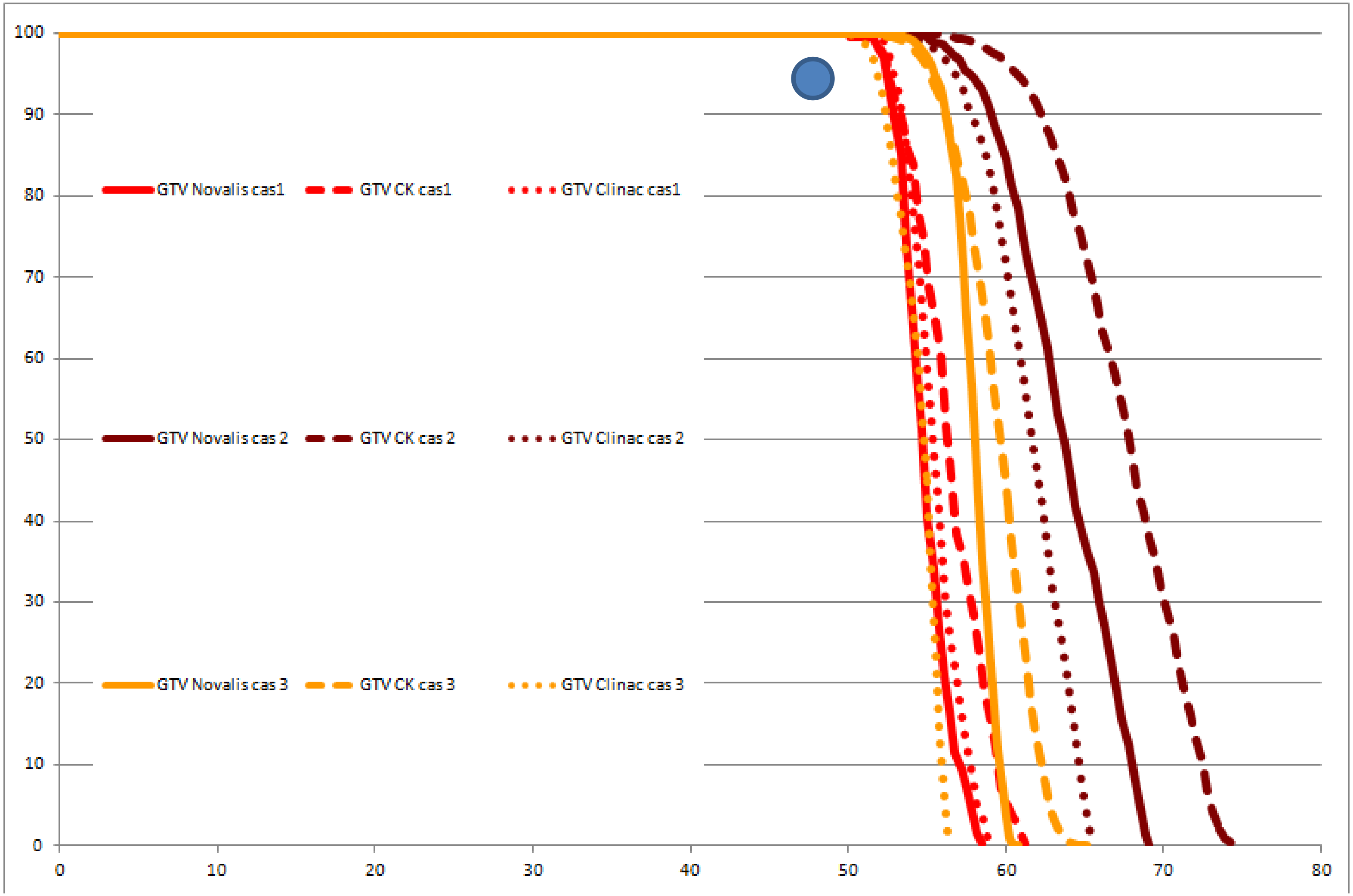

100
90
80
70
60
50
40
30
20
10
0
0
10
20
30
40
50
60
70
80
GTV Novalis cas1
GTV CK cas1
GTV Clinac cas1
GTV Novalis cas 2
GTV CK cas 2
GTV Clinac cas 2
GTV Novalis cas 3
GTV CK cas 3
GTV Clinac cas 3

# With a criteria on PTV

✓ doses to GTV are variables :

- Among cases
- For one case, among platforms

# If we prescribe on GTV median dose (D50%)

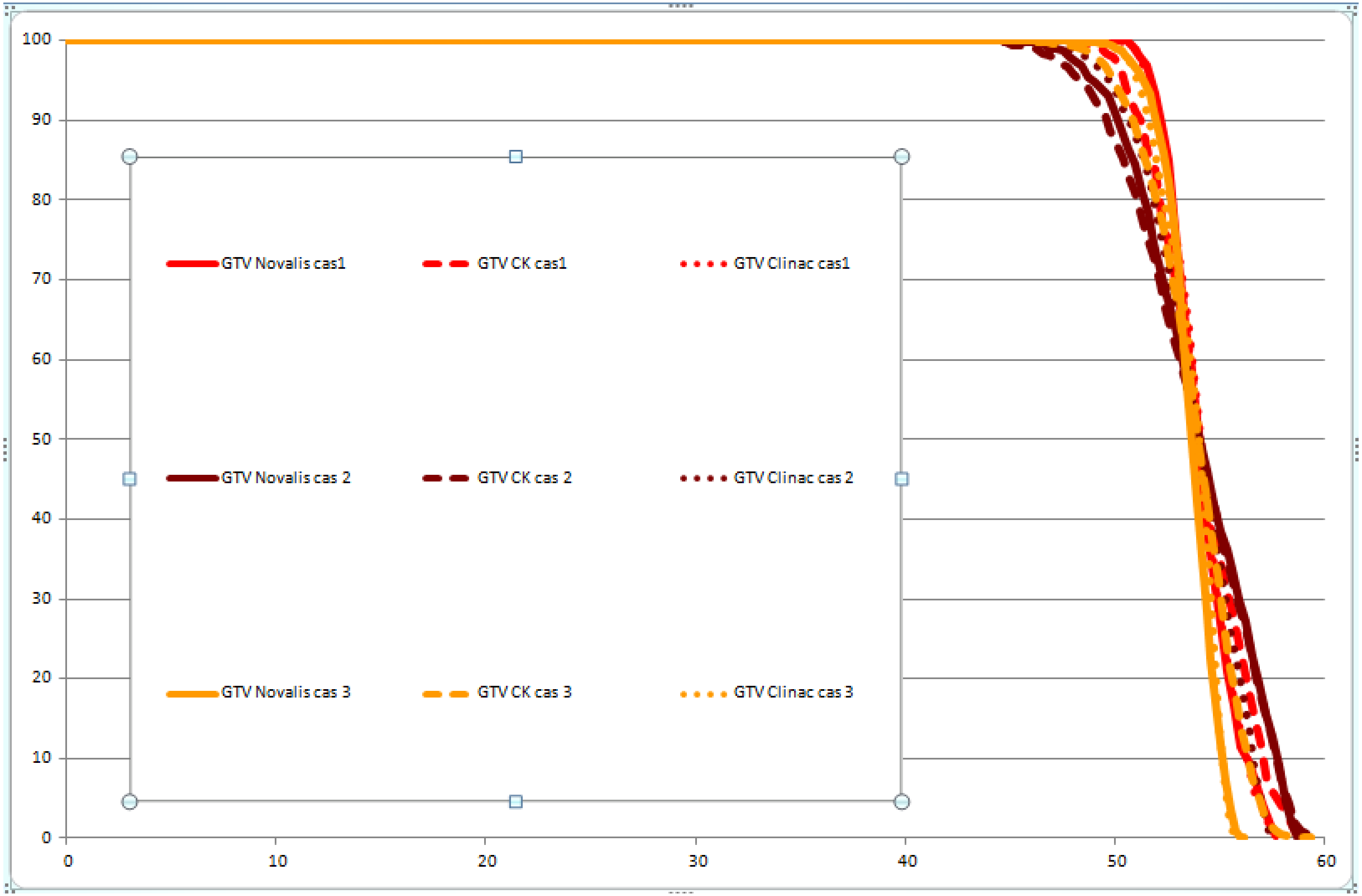

# Conclusion

- Use type B algorithms
- At least report GTV D50%, D98%
- We suggest to prescribe on GTV D50%
